# 2-[(4-Bromo­benzyl­idene)amino]­ethanol

**DOI:** 10.1107/S1600536812048155

**Published:** 2012-11-28

**Authors:** Vashen Moodley, Werner E. Van Zyl

**Affiliations:** a School of Chemistry and Physics, University of KwaZulu-Natal, Westville Campus, Private Bag X54001, Durban 4000, South Africa

## Abstract

In the crystal structure of the title compound, C_9_H_10_BrNO, molecules are linked *via* O—H⋯N hydrogen bonds of a moderate strength between the hy­droxy groups and the imine N atoms. These hydrogen bonds, as well as the planes of the bromo­phenyl rings, are situated in alternating planes parallel to (013) and (0-13). In addition, there are weak C—H⋯π inter­actions in the structure.

## Related literature

For previous work on the preparation of imine-based ligands, by our group, see: Williams *et al.* (2007 ▶). For related structures and their preparation, see: Elslager *et al.* (1956 ▶); Vennila *et al.* (2010 ▶); Jafarpour *et al.* (2011 ▶). For imines, see: Morrison *et al.* (1987 ▶); Tidwell (2007 ▶) and for their biological activity, see: Solomon & Lowery (1993 ▶); Fioravanti *et al.* (1995 ▶); Mallikarjun & Sangamesh (1997 ▶); Samadhiya & Halve (2001 ▶); Gerdemann *et al.* (2002 ▶); Veverková & Toma (2008 ▶); Khan *et al.* (2009 ▶). For classification of hydrogen bonds, see: Gilli & Gilli (2009 ▶).
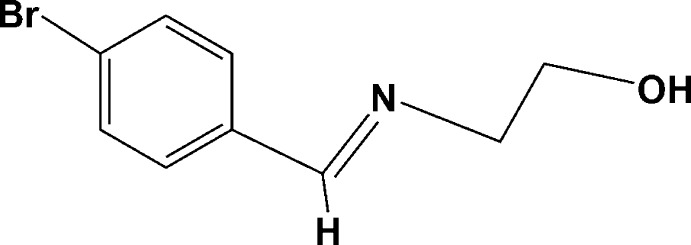


## Experimental

### 

#### Crystal data

C_9_H_10_BrNO
*M* *_r_* = 228.09
Monoclinic, 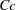

*a* = 22.349 (4) Å
*b* = 6.0328 (10) Å
*c* = 7.3673 (12) Å
β = 107.980 (3)°
*V* = 944.8 (3) Å^3^
*Z* = 4
Mo *K*α radiation
μ = 4.30 mm^−1^
*T* = 173 K
0.36 × 0.15 × 0.02 mm

#### Data collection

Bruker Kappa DUO APEXII diffractometer
Absorption correction: multi-scan (*SADABS*; Sheldrick, 2008*a* ▶) *T*_min_ = 0.305, *T*_max_ = 0.919
3713 measured reflections
1651 independent reflections
1550 reflections with *I* > 2σ(*I*)
*R*_int_ = 0.035

#### Refinement

*R*[*F* ^2^ > 2σ(*F* ^2^)] = 0.025
*wR*(*F* ^2^) = 0.059
*S* = 1.00
1651 reflections
113 parameters
1 restraint
H atoms treated by a mixture of independent and constrained refinement
Δρ_max_ = 0.71 e Å^−3^
Δρ_min_ = −0.45 e Å^−3^
Absolute structure: Flack (1983 ▶), 788 Friedel pairs
Flack parameter: 0.022 (12)

### 

Data collection: *APEX2* (Bruker, 2006 ▶); cell refinement: *SAINT* (Bruker, 2006 ▶); data reduction: *SAINT*; program(s) used to solve structure: *SHELXS97* (Sheldrick, 2008*b* ▶); program(s) used to refine structure: *SHELXL97* (Sheldrick, 2008*b* ▶); molecular graphics: *ORTEP-3* (Farrugia, 2012 ▶); software used to prepare material for publication: *SHELXL97*.

## Supplementary Material

Crystal structure: contains datablock(s) I, global. DOI: 10.1107/S1600536812048155/fb2276sup1.cif

Structure factors: contains datablock(s) I. DOI: 10.1107/S1600536812048155/fb2276Isup2.hkl

Supplementary material file. DOI: 10.1107/S1600536812048155/fb2276Isup3.cml

Additional supplementary materials:  crystallographic information; 3D view; checkCIF report

## Figures and Tables

**Table 1:** Hydrogen-bond geometry (Å, °) *Cg*1 is the centroid of the C1–C6 ring.

*D*—H⋯*A*	*D*—H	H⋯*A*	*D*⋯*A*	*D*—H⋯*A*
O1—H1⋯N1^i^	0.97 (3)	1.83 (3)	2.791 (4)	172 (4)
C2—H2⋯*Cg*1^ii^	0.95	2.83	3.58 (3)	137
C5—H5⋯*Cg*1^iii^	0.95	2.79	3.512 (3)	134

Symmetry codes: (i) 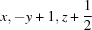; (ii) 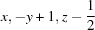; (iii) 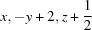.

**Fig. 1.**
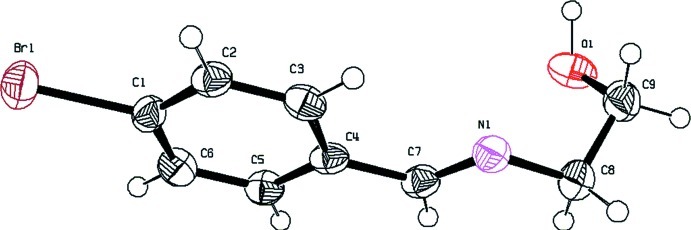
The molecule with the atom labelling scheme. The displacement ellipsoids are shown the 50% probability level.

**Fig. 2.**
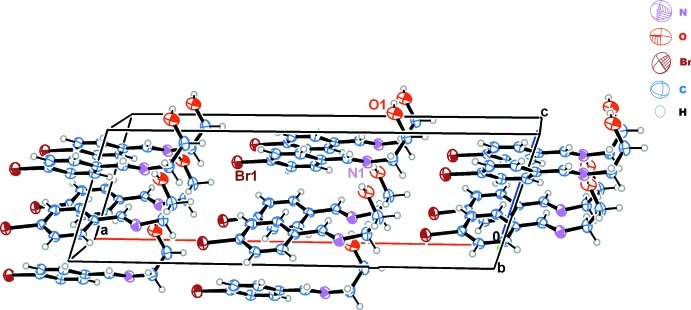
The crystal packing of the title compound as seen approximately along the b axis.

**Fig. 3.**
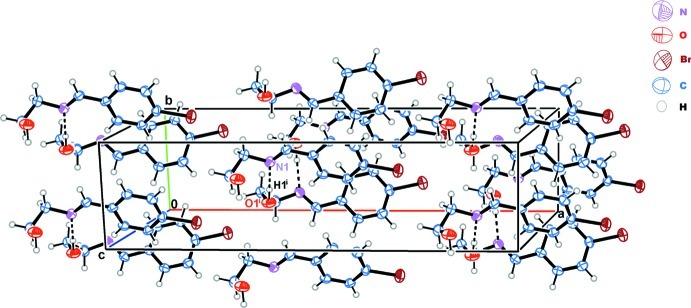
The crystal structure of the title compound with the hydrogen bonds (Tab. 1) indicated as dashed lines. The structure is approximately viewed along the c axis. Symmetry code: (i) x, -y + 1, z + 1/2.

## supplementary crystallographic information

### Comment

The title compound, 2-(4-bromobenzylideneamino)ethanol, was prepared and crystallized during our ongoing research on nitrogen-based ligands (Williams *et al.*, 2007). The molecule belongs to the class of compounds known as the Schiff bases (Tidwell, 2007). The primary feature of all the Schiff bases is presence of a functional group that includes a carbon-nitrogen double bond, known as the imine group. The imine's nitrogen atom can be connected to a hydrogen atom, or aryl or alkyl groups (Jafarpour *et al.*, 2011). In cases where the hydrogen atom is bonded to the nitrogen atom, *i. e.* RCH═NH, the imine is an unstable chemical species (an intermediate) because it is prone to decomposition (through reaction with oxygen or moisture in air). This intermediate is formed during the reaction between an aldehyde and ammonia that leads to direct conversion into the amine product by a process called reductive amination (Morrison *et al.*, 1987). In cases where an aryl- or alkyl group (as is the case in the present study) is bonded to the nitrogen atom, *i. e.* R C═NR, the imine shows a significant improvement in stability and the compound can be isolated. In the present study, the alkyl group with a stabilizing effect (specifically, an ethyl group) is bonded to the nitrogen atom while the ethyl group also contains a hydroxyl moiety.

The Schiff bases have a number of useful applications such as their use as ligands in coordination chemistry and their role in forming supramolecular metallocycles and metallocages. In addition, the aryl Schiff bases, such as the title compound, are also commonly synthesized for pharmaceutical objectives or used in the synthesis of biologically useful compounds. These biological compounds include a range of applications in anti-fungal, anti-inflammatory, anti-HIV, anti-bacterial, herbicidal, and anti-cancer activities (Khan *et al.*, 2009; Gerdemann *et al.*, 2002; Samadhiya & Halve, 2001; Mallikarjun & Sangamesh, 1997; Fioravanti *et al.*, 1995; Solomon & Lowery, 1993).

As a result of importance of the Schiff bases outlined above, new synthesis methods are being developed in order to obtain better yields at lower cost (Veverková & Toma, 2008).

The most commonly used procedure for the synthesis of the Schiff base compounds involves condensation of an amine with an aldehyde (Elslager *et al.*, 1956). This was also the procedure that we have used in the present study. We have synthesized the title molecule with goal to use it as a ligand in metal complexation. As a result, we wanted to discover its 3-dimensional spatial arrangement in the solid-state so that we can determine whether appropriate binding sites through donor N and O atoms on the title compound has the potential to bind to a metal center. This hypothesis needs to be tested with future experimentation.

The basic structural features of the title structure (Fig. 1) agrees well with the geometric parameters of similar previously synthesized structures such as (*E*)-4-[(4-bromobenzylidene)amino]-phenol (Vennila *et al.*, 2010). The C-Br bond distance in the title compound is 1.900 (3) Å, which is in agreement with the related value of 1.894 (2) Å (Vennila *et al.*, 2010). The packing of the molecules is shown in Figs. 2 and 3. Fig 3 shows the O-H···N hydrogen bonds of a moderate strength (Gilli & Gilli, 2009; Table 1) between the hydroxyls and the imine N atoms. The hydrogen bonds as well as

the planes of the bromophenyl rings are situated in alternating planes parallel to (0 1 3) and (0 -1 3). In addition, there are weak C-H···π-electron ring interactions in the structure (Table 1).

### Experimental

To a solution of 4-bromobenzaldehyde (2.031 g, 0.011 mol) in dry toluene (40 ml), ethanolamine (0.671 g, 0.011 mol), also dissolved in dry toluene (40 ml), was added dropwise in intervals of 30 minutes while stirring the formed suspension. The reaction mixture was stirred under ambient conditions for further 2 hrs, and then refluxed at 120 °C for 3 hrs. The water was removed by distillation in a Dean-Stark distillation receiver and the toluene was removed *in vacuo.* Upon cooling of the reaction mixture, the material precipitated. The viscous matter was dissolved in 20 ml of dichloromethane. The solution was then filtered through Celite (diatomaceous earth, a filter-aid) and dried over anhydrous magnesium sulfate to remove traces of water. Large, platelike colourless crystals with dimensions 0.36×0.15×0.02 mm and a hexagonal shape were grown from 200 mg of powder sample through slow diffusion of hexane solvent layered on top of dichloromethane solution. Yield: 1.516 g (60.4%); M.p. 81 - 82°C. ^1^H NMR: *δ* (p.p.m.): 3.69 (2*H*, t, *J* = 4.85 Hz, C*H_2_*OH), 3.86 (2*H*, d, *J* = 5.06 Hz, C*H_2_*-N), 7.51 (4*H*, dd, *J* = 6.24 Hz, 8.81 aryl-*H*), 8.61 (1*H*, s, C_H_═N). ^13^C NMR: *δ* (p.p.m.): 62.119 (CH_2_OH), 63.331 (N—CH_2_), 125.314 (*C*-aryl) 129.607 (*C*-aryl), 131.860 (*C*-aryl), 134.629 (*C*-aryl), 162.009 (C═N).

### Refinement

The hydrogen atoms were identified in the difference electron density map, after which the aryl and methyl H atoms were situated into idealized positions and constrained to ride on their parent atoms, with C···H = 0.95 and 0.98 Å, respectively. *U*_iso_(H_aryl_) = 1.2×*U*_eq_C_aryl_ and *U*_iso_(H_methyl_) = 1.5×*U*_eq_C_methyl_. The methyl groups were refined as rigid rotors in order to fit to the electron density. The positional parameters of the primary and the secondary amine H atoms were freely refined while their displacement parameters were constrained as 1.2 multiples of their carrier atoms. 788 Friedel pairs have been measured.

### Crystal data

**Table:**

C_9_H_10_BrNO	*F*(000) = 456
*M**_r_* = 228.09	*D*_x_ = 1.604 Mg m^−^^3^
Monoclinic, *C**c*	Mo *K*α radiation, λ = 0.71073 Å
Hall symbol: C -2yc	Cell parameters from 3713 reflections
*a* = 22.349 (4) Å	θ = 3.5–25.3°
*b* = 6.0328 (10) Å	µ = 4.30 mm^−^^1^
*c* = 7.3673 (12) Å	*T* = 173 K
β = 107.980 (3)°	Plate, colourless
*V* = 944.8 (3) Å^3^	0.36 × 0.15 × 0.02 mm
*Z* = 4	

### Data collection

**Table:**

Bruker Kappa DUO APEXII diffractometer	1651 independent reflections
Radiation source: fine-focus sealed tube	1550 reflections with *I* > 2σ(*I*)
Graphite monochromator	*R*_int_ = 0.035
0.5° φ scans and ω scans	θ_max_ = 25.3°, θ_min_ = 3.5°
Absorption correction: multi-scan (*SADABS*; Sheldrick, 2008*a*)	*h* = −24→26
*T*_min_ = 0.305, *T*_max_ = 0.919	*k* = −7→7
3713 measured reflections	*l* = −8→8

### Refinement

**Table:**

Refinement on *F*^2^	Secondary atom site location: difference Fourier map
Least-squares matrix: full	Hydrogen site location: difference Fourier map
*R*[*F*^2^ > 2σ(*F*^2^)] = 0.025	H atoms treated by a mixture of independent and constrained refinement
*wR*(*F*^2^) = 0.059	*w* = 1/[σ^2^(*F*_o_^2^) + (0.0174*P*)^2^] where *P* = (*F*_o_^2^ + 2*F*_c_^2^)/3
*S* = 1.00	(Δ/σ)_max_ < 0.001
1651 reflections	Δρ_max_ = 0.71 e Å^−^^3^
113 parameters	Δρ_min_ = −0.45 e Å^−^^3^
1 restraint	Absolute structure: Flack (1983), 788 Friedel pairs
37 constraints	Flack parameter: 0.022 (12)
Primary atom site location: structure-invariant direct methods	

### Special details

**Table:**

Geometry. All e.s.d.'s (except the e.s.d. in the dihedral angle between two l.s. planes) are estimated using the full covariance matrix. The cell e.s.d.'s are taken into account individually in the estimation of e.s.d.'s in distances, angles and torsion angles; correlations between e.s.d.'s in cell parameters are only used when they are defined by crystal symmetry. An approximate (isotropic) treatment of cell e.s.d.'s is used for estimating e.s.d.'s involving l.s. planes.
Refinement. Refinement of *F*^2^ against ALL reflections. The weighted *R*-factor *wR* and goodness of fit *S* are based on *F*^2^, conventional *R*-factors *R* are based on *F*, with *F* set to zero for negative *F*^2^. The threshold expression of *F*^2^ > σ(*F*^2^) is used only for calculating *R*-factors(gt) *etc*. and is not relevant to the choice of reflections for refinement. *R*-factors based on *F*^2^ are statistically about twice as large as those based on *F*, and *R*- factors based on ALL data will be even larger.

### Fractional atomic coordinates and isotropic or equivalent isotropic displacement parameters (Å^2^)

**Table:**

	*x*	*y*	*z*	*U*_iso_*/*U*_eq_	
Br1	0.682260 (18)	0.64650 (5)	0.71855 (2)	0.04348 (12)	
O1	0.34787 (17)	0.6979 (5)	1.1065 (4)	0.0461 (7)	
H1	0.359 (2)	0.554 (4)	1.165 (7)	0.069*	
N1	0.37448 (13)	0.7338 (5)	0.7440 (4)	0.0352 (7)	
C1	0.60169 (15)	0.7091 (5)	0.7424 (4)	0.0304 (7)	
C2	0.55354 (15)	0.5562 (6)	0.6774 (4)	0.0302 (7)	
H2	0.5610	0.4194	0.6244	0.036*	
C3	0.49499 (17)	0.6038 (5)	0.6903 (5)	0.0315 (7)	
H3	0.4620	0.4989	0.6463	0.038*	
C4	0.48338 (18)	0.8054 (6)	0.7676 (5)	0.0287 (8)	
C5	0.53236 (16)	0.9557 (6)	0.8318 (4)	0.0315 (7)	
H5	0.5249	1.0929	0.8844	0.038*	
C6	0.59188 (17)	0.9099 (6)	0.8208 (5)	0.0347 (8)	
H6	0.6252	1.0134	0.8658	0.042*	
C7	0.42189 (16)	0.8625 (5)	0.7886 (5)	0.0310 (7)	
H7	0.4173	1.0043	0.8386	0.037*	
C8	0.31685 (17)	0.8097 (6)	0.7782 (6)	0.0387 (8)	
H8A	0.2815	0.8034	0.6578	0.046*	
H8B	0.3220	0.9656	0.8224	0.046*	
C9	0.30217 (18)	0.6660 (6)	0.9269 (6)	0.0404 (8)	
H9A	0.2600	0.7043	0.9354	0.048*	
H9B	0.3016	0.5083	0.8893	0.048*	

### Atomic displacement parameters (Å^2^)

**Table:**

	*U*^11^	*U*^22^	*U*^33^	*U*^12^	*U*^13^	*U*^23^
Br1	0.03404 (18)	0.0539 (2)	0.04599 (18)	0.0060 (3)	0.01748 (12)	0.0006 (3)
O1	0.067 (2)	0.0296 (13)	0.0442 (18)	−0.0032 (15)	0.0204 (15)	−0.0002 (12)
N1	0.0358 (17)	0.0331 (16)	0.0399 (16)	0.0043 (13)	0.0164 (12)	0.0035 (13)
C1	0.0268 (18)	0.0386 (18)	0.0265 (16)	0.0057 (14)	0.0093 (13)	0.0052 (14)
C2	0.0363 (19)	0.0256 (16)	0.0297 (16)	0.0036 (14)	0.0114 (14)	0.0000 (14)
C3	0.040 (2)	0.0247 (16)	0.0302 (16)	−0.0039 (14)	0.0115 (14)	−0.0020 (13)
C4	0.038 (2)	0.0261 (16)	0.0236 (17)	0.0054 (16)	0.0112 (15)	0.0020 (13)
C5	0.039 (2)	0.0265 (18)	0.0300 (17)	0.0017 (15)	0.0117 (14)	−0.0028 (15)
C6	0.037 (2)	0.0335 (17)	0.0329 (17)	−0.0057 (15)	0.0094 (14)	−0.0030 (15)
C7	0.0360 (19)	0.0284 (17)	0.0301 (16)	0.0067 (14)	0.0125 (14)	0.0021 (13)
C8	0.0314 (19)	0.0397 (18)	0.047 (2)	0.0041 (15)	0.0153 (15)	0.0024 (16)
C9	0.036 (2)	0.0363 (18)	0.054 (2)	0.0014 (15)	0.0212 (17)	−0.0019 (17)

### Geometric parameters (Å, º)

**Table:**

Br1—C1	1.900 (3)	C4—C5	1.387 (5)
O1—C9	1.413 (5)	C4—C7	1.471 (5)
O1—H1	0.968 (5)	C5—C6	1.385 (5)
N1—C7	1.272 (4)	C5—H5	0.9500
N1—C8	1.461 (4)	C6—H6	0.9500
C1—C2	1.386 (5)	C7—H7	0.9500
C1—C6	1.388 (5)	C8—C9	1.510 (5)
C2—C3	1.371 (5)	C8—H8A	0.9900
C2—H2	0.9500	C8—H8B	0.9900
C3—C4	1.401 (5)	C9—H9A	0.9900
C3—H3	0.9500	C9—H9B	0.9900
			
C9—O1—H1	108 (3)	C5—C6—H6	120.8
C7—N1—C8	118.2 (3)	C1—C6—H6	120.8
C2—C1—C6	121.3 (3)	N1—C7—C4	124.1 (3)
C2—C1—Br1	119.5 (3)	N1—C7—H7	117.9
C6—C1—Br1	119.2 (3)	C4—C7—H7	117.9
C3—C2—C1	119.5 (3)	N1—C8—C9	110.2 (3)
C3—C2—H2	120.2	N1—C8—H8A	109.6
C1—C2—H2	120.2	C9—C8—H8A	109.6
C2—C3—C4	120.7 (3)	N1—C8—H8B	109.6
C2—C3—H3	119.7	C9—C8—H8B	109.6
C4—C3—H3	119.7	H8A—C8—H8B	108.1
C5—C4—C3	118.7 (3)	O1—C9—C8	110.2 (3)
C5—C4—C7	118.4 (3)	O1—C9—H9A	109.6
C3—C4—C7	122.8 (3)	C8—C9—H9A	109.6
C6—C5—C4	121.4 (3)	O1—C9—H9B	109.6
C6—C5—H5	119.3	C8—C9—H9B	109.6
C4—C5—H5	119.3	H9A—C9—H9B	108.1
C5—C6—C1	118.5 (3)		
			
C6—C1—C2—C3	0.1 (5)	C2—C1—C6—C5	−0.5 (5)
Br1—C1—C2—C3	−178.4 (2)	Br1—C1—C6—C5	178.1 (2)
C1—C2—C3—C4	0.2 (5)	C8—N1—C7—C4	178.1 (3)
C2—C3—C4—C5	−0.3 (5)	C5—C4—C7—N1	−176.3 (3)
C2—C3—C4—C7	−178.6 (3)	C3—C4—C7—N1	1.9 (5)
C3—C4—C5—C6	0.0 (5)	C7—N1—C8—C9	−115.0 (4)
C7—C4—C5—C6	178.3 (3)	N1—C8—C9—O1	67.6 (4)
C4—C5—C6—C1	0.4 (5)		

### Hydrogen-bond geometry (Å, º)

Cg1 is the centroid of the C1–C6 ring.

**Table:**

*D*—H···*A*	*D*—H	H···*A*	*D*···*A*	*D*—H···*A*
O1—H1···N1^i^	0.97 (3)	1.83 (3)	2.791 (4)	172 (4)
C2—H2···*Cg*1^ii^	0.95	2.83	3.58 (3)	137
C5—H5···*Cg*1^iii^	0.95	2.79	3.512 (3)	134

Symmetry codes: (i) *x*, −*y*+1, *z*+1/2; (ii) *x*, −*y*+1, *z*−1/2; (iii) *x*, −*y*+2, *z*+1/2.
